# Hesitancy toward Childhood Vaccinations: Preliminary Results from an Albanian Nursing Staff's Investigation

**DOI:** 10.1155/2022/7814488

**Published:** 2022-09-09

**Authors:** Enkeleda Gjini, Mariachiara Carestia, Fabian Cenko, Daniele Di Giovanni, Irsida Mehmeti, Stefania Moramarco, Alban Yulli, Ersilia Buonomo

**Affiliations:** ^1^Faculty of Medicine, Catholic University of “Our Lady of Good Counseil”, Tirane, Albania; ^2^Department of Biomedicine and Prevention, University of Rome Tor Vergata, Rome, Italy; ^3^Unicamillus-Saint Camillus International University of Health Sciences, Rome, Italy; ^4^Department of Industrial Engineering, University of Rome Tor Vergata, Rome, Italy; ^5^Faculty of Pharmacy, Catholic University of “Our Lady of Good Counseil”, Tirane, Albania; ^6^Faculty of Medicine, Tirana Medical University, Tirane, Albania

## Abstract

Healthcare professionals are important models for their patients since their individual knowledge and attitudes toward vaccination can influence the patient's willingness to adhere to vaccination campaigns. After developing a structured questionnaire, it was administered to a sample of nursing staff working in public vaccination centers in Albania (December 2020-January 2021), in order to conduct a preliminary investigation aimed at describing knowledge, attitudes, beliefs, and hesitancy toward childhood vaccinations. Among the sample of nurses involved in the administration of vaccines (n.64, 92% females), most of them were confident about vaccines and favorable to childhood vaccinations (90%). However, when specifically investigating beliefs, nearly a quarter of the sample showed to be hesitant; 22% were unsure or partially agreed that vaccines might cause conditions such as autism and multiple sclerosis. A high risk of hesitancy was identified in the youngest staff especially when their work experience was below 10 years or when they graduated less than 10 years before (OR: 5.3, CI: 1.4–19.5; and OR: 4.2 CI: 1.2–14.6). Similarly, a low acceptance rate (54%) was detected for future childhood SARS-CoV-2 vaccines among the nurses, which is a sign of high levels of vaccine hesitancy. With regard to knowledge about childhood vaccine contraindications, none of the nurses identified all the ten correct answers, while only 13% answered at least six questions correctly. These preliminary results highlight the need of investigating more Albanian nursing staff's knowledge and attitudes toward child vaccinations, therefore investing in tailored training. Due to the ongoing Covid-19 pandemic and the roll-out of mass vaccination, the role of healthcare workers remains crucial and needs more support to manage the changing public opinion as well as quickly evolving vaccine technologies.

## 1. Background

Vaccination is one of the most effective ways of controlling infectious diseases, particularly in the pandemic era. However, vaccine hesitancy, the delay in acceptance or refusal of vaccines despite the availability of vaccination services, has become a growing concern globally [1]. Risk perceptions and concerns about vaccine safety, attitudes, inadequate or poor communication about vaccines, social and cultural norms, and structural barriers could all be associated with vaccine hesitancy [2]. Therefore, vaccine hesitancy is a complex and context-specific issue, with key reasons behind it defined as complacency, inconvenience, and lack of confidence [3].

The WHO recognized vaccine hesitancy as one of the top ten public health concerns and threats to global health in 2019 since it is one of the reasons for vaccination coverage decreasing all over the world [4]. To determine the rate of vaccine hesitancy across the globe, three years of available data (2014–2016) were reviewed from the WHO/UNICEF joint report form (JRF), showing that the number of countries that reported “no vaccine hesitancy” was globally very low (from 6 to 7%) [5]. In addition, the large study conducted between November 2015 and December 2019 by De Figueiredo et al. reported that confidence in vaccines is still a concern particularly high in Europe where vaccine confidence has been persistently low since 2015 compared with other continents. However, in the same report, some signs of an increase in vaccine confidence, despite slow, were noted in the most recent years in some EU member states [6].

Since healthcare professionals are important role models for their patients, their function is crucial in delivering recommendations based on scientific evidence and increasing public awareness about the benefits of immunization. Their individual perceptions, knowledge, and attitudes can influence the family's decision to vaccinate their children. Therefore, it is essential to ensure that healthcare providers are aware of the characteristics, safety, and efficacy of vaccines [7]. Given the Strategic Objective n.2 of the Global Vaccine Action Plan, vaccine hesitancy surveys among health staff are crucial to promptly identify, understand, and address major determinants of vaccine hesitancy within different communities [8]. However, research in this area is still insufficient, especially in low-income and middle-income countries. This study aims at describing the preliminary findings about knowledge, attitudes, and beliefs of Albanian healthcare workers involved in the administration of childhood vaccines, therefore feeding the scientific literature with more evidence on nursing staff's confidence and hesitancy toward well-known childhood vaccination in Albania.

## 2. Methods

### 2.1. Study Design and Sample

Between December 2020 and January 2021, we conducted an observational cross-sectional study consisting of data collected through a structured survey on a convenience sample of nurses working in public vaccination centers covering the majority of regions in Albania. This survey was a pilot study conducted during the Covid-19 pandemic, and the Albanian Ministry of Health indicated the main 14 centers active in childhood vaccination in order to represent north, central, and south regions, as well as urban and rural catchment areas.

All the nurses of every selected health center were asked to answer the self-administrated questionnaire. Before the survey, informed consent was obtained from all the participants.

In this report, we discuss only the preliminary findings of a survey among healthcare workers, which was approved by the Ethical Committee of the Ministry of Health and Social Protection of Albania (n.303/46 of October 16, 2020).

### 2.2. Questionnaire

After reviewing the literature [9, 10], we developed a semistructured questionnaire consisting of four sections:

- Nurses' general information, including years of practice and years since graduation
- Nurses' beliefs, attitudes, confidence, and hesitancy toward vaccination topics were assessed with the support of a questionnaire composed of 14 statements (according to a five-point Likert scale)
- Nurses' perceived impact of different training tools in obtaining knowledge on pediatric vaccination
- To evaluate their knowledge regarding vaccine contraindications, they were asked to classify 10 clinical conditions as contraindications or not
- To evaluate hesitancy about the SARS-CoV-2 vaccine in children, the question asked was “If a pediatric SARS-CoV-2 vaccine was available would you vaccinate your patients?”

The questionnaire was self-administrated. Participants were informed about the aim of the study and the usage of data. They were assured confidentiality and anonymity by using a codified number to identify each respondent. The respondents could not consult any material or each other when filling in the questionnaire.

### 2.3. Statistical Analysis

Data collected have been presented as numbers and percentages. The statistical elaboration of the data was performed using IBM SPSS Statistics (version 26). Odds ratios (ORs) were calculated with their 95% confidence interval (95% CI) in order to investigate factors that could be associated with an increased risk of vaccine hesitancy. When referring to knowledge regarding vaccine contraindications, the median incorrect answers were reported as for the total sample, then differences between answers' scores of hesitant vs. not hesitant nurses were analyzed by using the nonparametric Mann–Whitney *U* test. Differences between the number of questions correctly answered based on having 10 years (or having less) of working experience or having graduated 10 years (or less) from the survey were also investigated using the nonparametric Mann–Whitney *U* test.

## 3. Results

**Figure 1:**
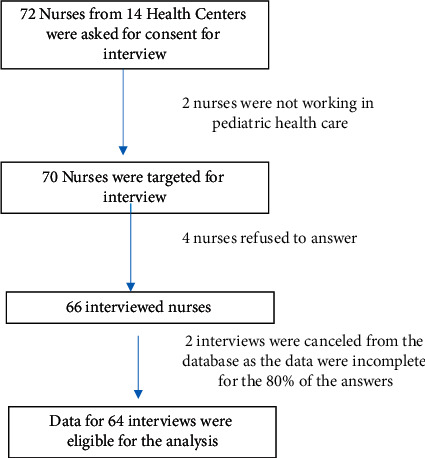
Flowchart of the health staff enrolled.

Data from 64 Albanian nurses (92.2% females) were eligible for the analysis. The questionnaires covered 88.9% of the overall nursing staff of the centers.  Figure 1 shows details of the sample.

Among the responders, 61% (n.39) were less than 45 years old; 93.8% (n.60) were working in a vaccination center located in urban areas (37.5% in Tirana, 17.2% in Elbasan, 12.5% in Vlora, 10.9% in Durres, 7.8% in Shkodra, and 7.8% in Lezha), while 6.3% (n.4) of them were in the rural area (Kruja).

Among the nurses, 62.5% (n.40) of them graduated more than ten years before the survey took place (including 9.4% of whom graduated more than twenty years before) and 37.5% (n.24) less than 10 years; 40.6% (n.26) were working in vaccination centers since less than 10 years and 59.4% (n.38) since more than 10 years (including 17.3% since more than 20 years).

When asked to rate their level of agreement with negative statements about vaccine safety and effectiveness (Table 1), nurses agreed completely or partially on “Vaccines weaken or overload the immune system” (nearly 18%), “Children receive too many vaccines” (nearly 8%), “Childhood vaccines are given too early” (more than 18%), and “It is better for children to develop natural immunity rather than to get a vaccine” (nearly 16%). Lower confidence was observed concerning the statement “Conditions such as autism and multiple sclerosis may be caused by vaccines” since 17.5% of the sample showed to be hesitant (unsure, partially agree, or fully agree) about it.

Conversely, nearly 75% of the sample agreed with both statements “Vaccines are among the safest and most tested medicinal products” and “Vaccine information provided by health authorities and scientific societies is reliable.” When considering vaccines' cost-effectiveness, confident responders lowered to 42%.

**Figure 2:**
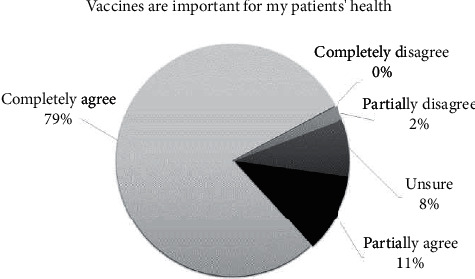
Confidence toward child vaccinations.

Despite a substantial proportion of nurses showed hesitancy about specific vaccination aspects, most of them still reported high general vaccine confidence, with 90% fully or partially agreeing to the statement “Vaccinations are important for my patients' health” (Figure 2) and 84% fully or partially agreeing to the statement “When children get vaccinated, the whole community benefits.”

When asked about training in the last five years, 34.4% (n.22) of nurses declared to have attended vaccine courses or conferences, while 28.1% (n.18) had never attended any training courses on vaccine topics. Having less than 10 years of work experience increased the risk for hesitancy (OR 5.3 CI: 1.4–19.5) compared to those with 10 or more years of experience. Similarly, hesitancy was significantly higher among nurses who graduated during 10 years before the survey (OR 4.2 CI: 1.2–14.6) compared to nurses who had graduated 10 or more years before.

**Figure 3:**
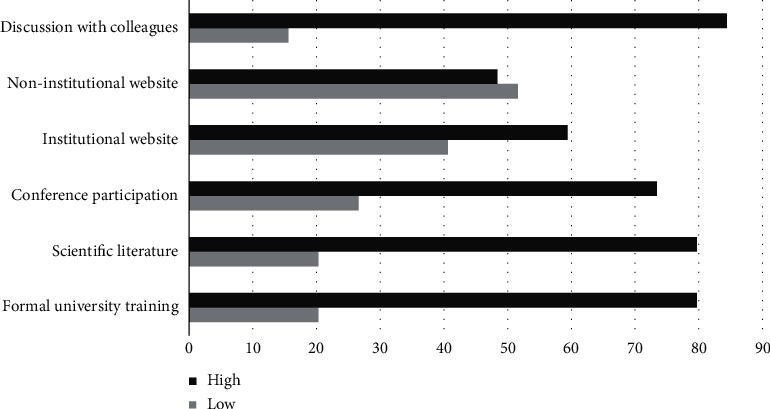
Self-perceived influence of different training tools in vaccine knowledge development.

Figure 3 shows the degree of self-perceived influence for different sources of information. High influence was attributed to the role of peer education: the most frequently reported influential source of knowledge on vaccines was the discussions with colleagues (84%). Nearly 80% of the sample considered formal university training of high importance, at the same level as scientific literature, followed by conference participation (73.4%) and institutional websites (59.4%). On the other hand, nearly half of the responders considered noninstitutional websites also of high importance (48.4%).

**Figure 4:**
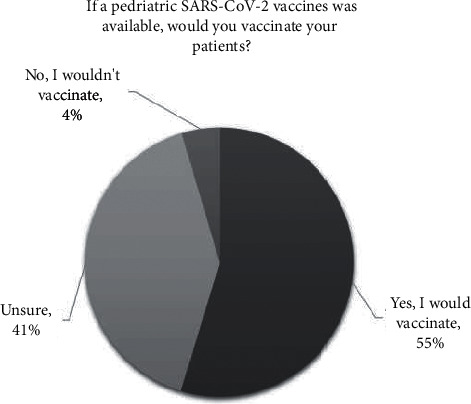
The acceptance rate of future SARS-CoV-2 pediatric vaccination.

Because the questionnaire was submitted during the pandemic of Covid-19 just before the first SARS-CoV-2 vaccine authorization, the authors added a specific question: “If a pediatric SARS-CoV-2 vaccine was available, would you vaccinate your patients?.” To that question, 35 nurses (54.7%) answered “yes” without hesitancy, 26 (40.6%) answered “I do not know,” and only 3 (4.6%) were completely hesitant, answering that they would have refused to vaccinate children (Figure 4).

Nurses were also asked to classify 10 clinical conditions or situations related to administering hexavalent vaccines as false contraindications, temporary contraindications, or permanent contraindications. Specifically, Table 2 reports correct answers to the question “Your patient is scheduled to receive the second dose of Hexavalent vaccines. Which of the following conditions do you consider contraindicated?.”

None of the nurses identified all the ten correct answers about contraindications to the hexavalent vaccine. Only 13% answered correctly to at least six questions. The median number of questions correctly answered was 4 (interquartile range 3.0), with no differences for years of working experience (*p* = 0.9) and years from graduation (*p* = 0.8). When dividing the sample into hesitant vs. not hesitant (n.14 hesitant: median 1.5, interquartile range 5.5; n.50 not hesitant: median 4, interquartile range 2.0), the difference between correct answer score was not statistically significant.

## 4. Discussion

Healthcare professionals, especially when employed in immunization delivery services in the primary healthcare sector, should acquire specific competencies in order to ensure that vaccines are provided to all those who need them in a safe and effective manner. Nurses in children's vaccination centers could play a key role in organizing and promoting immunization programs. Receiving information on knowledge gaps is of significant value for further management and development of focused educational programs [11].

In a study on vaccine confidence conducted in 2018 across 28 countries of the European Union, it was observed that even countries with well-established vaccination programs and high levels of confidence were not immune to rising vaccine hesitancy [12]. Despite the 2020 report's update showing growing confidence compared to the previous results, data confirmed that many Eastern European countries still rank particularly low in terms of their confidence in the safety, importance, and effectiveness of vaccines. These results show that rebuilding trust requires a long time and continuous efforts [9].

Few studies reporting healthcare workers' knowledge and practices toward vaccinations already exist in Albania [13, 14]. In our study, although most nurses were favorable to childhood vaccinations, we identified some associations between training gaps and overall attitudes towards vaccinations and/or specific knowledge and/or beliefs. In addition, in our analysis, we found a significant association between higher vaccine hesitancy attitudes and the youngest generations. As a matter of fact, a higher risk of being hesitant was identified among nurses with less than 10 years of work experience or those who graduated less than 10 years before the survey. Despite we acknowledge that other factors might have influenced these results, it is relevant to underline that nearly 30% of the survey participants referred that they never received training in vaccinology nor they attended conferences or vaccine courses in the last 5 years. It seems that vaccination training has not been a priority of continuous medical education in the Albanian health system at least in recent years. These results point out that a decline in vaccination knowledge might be related to declines in the education level, with the possibility of further deterioration in the future. The interaction between hesitancy and knowledge of healthcare workers is important since it improves adherence to the routine immunization programme for children [15].

These preliminary findings in Albanian nursing staff underline the need for further and detailed evaluation of knowledge gaps and vaccine hesitancy among health staff, in order to put the basis for continuous training and ongoing education on child vaccinations. This is particularly important in the case of instructing nurses about vaccine contraindications. Since the health personnel dedicated to vaccination services is essential for educating parents, they need to be equipped with appropriate skills and knowledge to address parents' vaccine hesitancy [16] and assure high vaccination coverage rates in the communities.

Furthermore, the low acceptance rate of future childhood SARS-CoV-2 vaccines among nurses demonstrates that vaccine hesitancy in healthcare workers remains a barrier to full population protection also in the context of the Covid-19 pandemic. These findings were in line with the hesitancy reported in nurses (45.5%) in another study conducted in Iraq on the health staff's willingness of receiving the Covid-19 vaccine [17] and with a recent survey among healthcare workers in Israel (55%) [18]. Similarly, another study by Aoun et al. conducted in the Middle East investigating healthcare staff attitudes toward any future COVID-19 vaccine found that the total rate of vaccine hesitancy in nurses was 63.2% [19]. All the three mentioned studies revealed that vaccine acceptance among nurses was significantly higher than among physicians, with fear of side effects followed by lack of confidence/information being the most common perceived barriers reported by Alhanabadi et al. and by Aoun et al.; however, these differences and these reasons were not investigated in the present study.

These results underline the need for stepping up immunization training campaigns for the health staff in Albania in the roll-out of mass vaccination campaigns, in order to fill current gaps and improve vaccination coverage in the future.

### 4.1. Limitations

We acknowledge that our results should be interpreted in the context of several limitations. First of all, this was a pilot study, and we analyzed data of a convenience small sample including health centers suggested by the Albanian Ministry of Health that were active at the moment of the survey since the ongoing pandemic of Covid-19. The small sample allowed us to perform only a crude analysis; therefore, other factors contributing to the vaccine hesitancy might have not been investigated.

Being a voluntary survey, only nurses from public health centers who willingly participated were included. We are therefore unaware if those who did not wish to participate held more vaccine doubts or not. The self-reported evaluations may be subject to expectancy bias and complacency bias. In addition, few questions were not answered..

Nevertheless, we would like to emphasize that this is one of the first studies that addresses this issue in the country, and it was aimed in the future to interview the entire population of professionals in public vaccination centers.

## Figures and Tables

**Table 1:** Nurses' beliefs and confidence toward vaccination.

Questionnaire statements	Completely disagree		Partially disagree		Unsure		Partially agree		Completely agree	
	*n*	%	*n*	%	*n*	%	*n*	%	*n*	%
Vaccines weaken or overload the immune system^	43	71.7	3	5.0	3	5.0	6	10.0	5	8.3
It is better for children to develop natural immunity by getting sick rather than to get a vaccine°	40	64.5	7	11.3	5	8.1	10	16.1	0	0.0
Healthy children do not need to be vaccinated	58	90.6	2	3.1	1	1.6	3	4.8	0	0
Conditions such as autism and multiple sclerosis may be caused by vaccines	49	77.8	3	4.8	10	15.9	1	1.6	0	0.00
Allergies are on the rise because of vaccinations	51	79.7	2	3.1	9	14.1	0	0	2	3.1
I am afraid that one of my patients may develop a severe adverse reaction following vaccination^*∗*^	13	20.6	10	15.9	15	23.8	19	30.2	6	9.5
Children receive too many vaccines	49	76.6	6	9.4	4	6.2	2	3.1	3	4.7
Vaccine policy is influenced by the financial profits of pharmaceutical companies°	33	53.2	10	16.1	11	17.7	6	9.8	2	3.2
Childhood vaccines are given too early^*∗*^	39	61.9	7	11.1	5	7.9	3	4.9	9	14.3
The frequency of adverse reactions to vaccines is underestimated^	38	63.3	6	10.0	12	20.0	3	5.0	1	1.7
Vaccination is cost-effective°	17	27.4	8	12.9	11	17.7	9	14.6	17	27.4
Vaccine information provided by health authorities and scientific societies is reliable	2	3.1	6	9.4	8	12.5	11	17.2	37	57.8
Vaccines are among the safest and most tested medicinal products^*∗*^	1	1.6	5	7.9	9	14.3	11	17.5	37	58.7
The second dose of MMR is useful	1	1.6	1	1.6	9	14.1	9	14.1	44	68.6
When children get vaccinated, the whole community benefits	2	3.1	2	3.1	6	9.4	6	9.4	48	75.0

°2 not responder, ^4 not responder, and ^*∗*^1 not responder.

**Table 2:** Correct responses on 10 clinical conditions or contraindications to administering the hexavalent vaccine.

Your patient is scheduled to receive the second dose of hexavalent vaccines. Which of the following conditions do you consider contraindicated?	Correct answer	Nurses answering correctly *n* (%)
Severe allergic reactions to a previous dose including anaphylaxis	Permanent contraindication	39 (60.9)
Fever following a previous dose	False contraindication	25 (39.1)
Acute severe gastroenteritis	Temporary contraindication	22 (34.4)
Otitis media without fever	False contraindication	17 (26.6)
Family history of adverse reactions following a pertussis vaccine dose	False contraindication	28 (43.8)
Acute upper airway infection without fever	False contraindication	14 (21.9)
History of mumps	False contraindication	27 (42.2)
Diagnosis of epilepsy well controlled	False contraindication	22 (34.4)
Fever 38–40° and moderate illness	Temporary contraindication	34 (53.1)
Fever >40° and severe illness	Temporary contraindication	19 (29.7)

## Data Availability

The dataset used to support the findings of this study is available from the corresponding author upon reasonable request.
